# Secondary pulmonary hypertension in an adult patient with Ebstein anomaly post-atrial septal defect closure; can it be reversible? A case report

**DOI:** 10.1093/ehjcr/ytaf527

**Published:** 2025-10-14

**Authors:** Ahmed Kheiwa, Vandad Saadat, Joseph Chung, Stephen Nageotte, Anees Razzouk

**Affiliations:** Department of Internal Medicine, Division of Cardiology, Loma Linda University Medical Center, 11234 Anderson St., Loma Linda, CA 92354, USA; Department of Internal Medicine, Loma Linda University Medical Center, 11234 Anderson St., Loma Linda, CA 92354, USA; Department of Internal Medicine, Division of Cardiology, Loma Linda University Medical Center, 11234 Anderson St., Loma Linda, CA 92354, USA; Departments of Pediatrics, Pediatric Cardiology, Loma Linda University Medical Center, 11234 Anderson St., Loma Linda, CA 92354, USA; Department of Cardiothoracic Surgery, Loma Linda University Medical Center, 11234 Anderson St., Loma Linda, CA 92354, USA

**Keywords:** Atrial septal defect (ASD), Atrialized right ventricle (aRV), Case report, Ebstein Anomaly (EA), Left pulmonary artery (LPA), Pulmonary hypertension (PHTN), Right atrium (RA), Right pulmonary artery (RPA), Right pulmonary veins (rPVs), Right ventricle (RV)

## Abstract

**Background:**

Pulmonary hypertension is often a difficult to diagnose condition, in particular in younger population of patients. Early diagnosis and treatment of this condition is crucial to prevent further morbidity and mortality.

**Case summary:**

A 32-year-old woman with a history of Ebstein anomaly (EA) and secundum atrial septal defect (ASD), who underwent transcatheter ASD closure at age 19, presented with progressive fatigue and exertional dyspnoea. Further evaluation revealed presence of compression of right pulmonary veins (rPVs) by the ASD device resulting in post-capillary pulmonary hypertension (PHTN) and worsening of right ventricular (RV) failure. Following surgical explantation of the ASD device, PHTN resolved with improvement of patient’s functional status.

**Discussion:**

This case depicts a rare haemodynamic entity of worsening PHTN in a 32-year-old patient with EA post-ASD closure. It illustrates a rare complication of compression of rPVs by the ASD device. Therefore, suspicion should be high for evaluating rPVs in EA with worsening PTHN and RV failure post-ASD closure.

Learning points

- Understand the signs, symptoms and physical exam findings of Ebstein anomaly.
- Recognize the potential complication of pulmonary vein compression as a complication of the percutaneous closure of atrial septal defect.

## Introduction

Secundum atrial septal defect (ASD) is a common lesion associated with Ebstein anomaly (EA). Although transcatheter closure is feasible, the decision to close ASD in EA is challenging and requires careful evaluation.

## Summary figure

**Figure:**
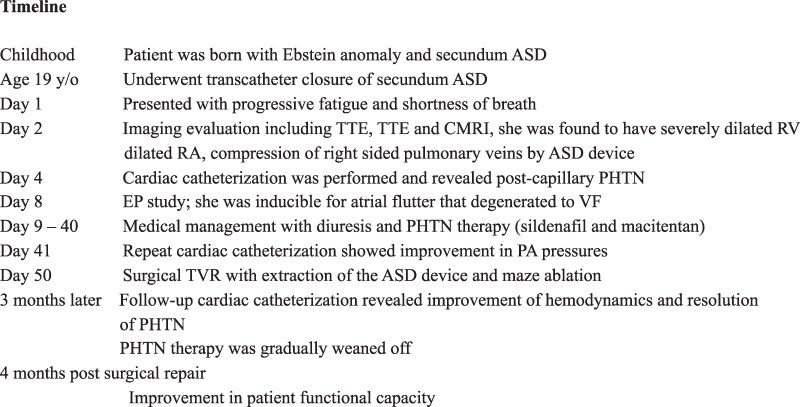


## Case presentation

**Figure 1:**
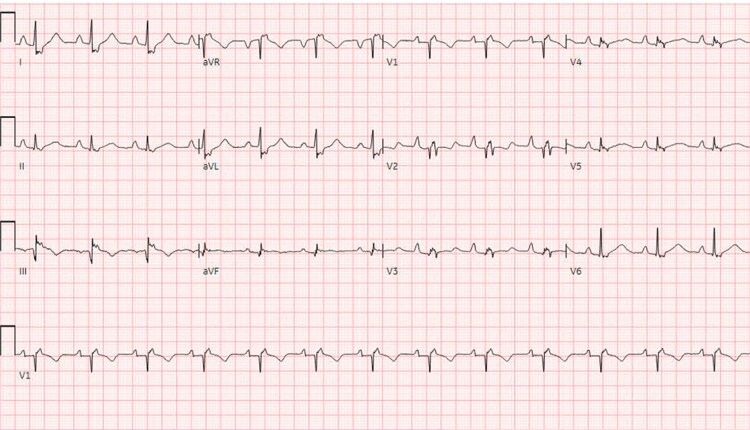
Electrocardiogram showing normal sinus rhythm with non-specific ST changes.

**Figure 2:**
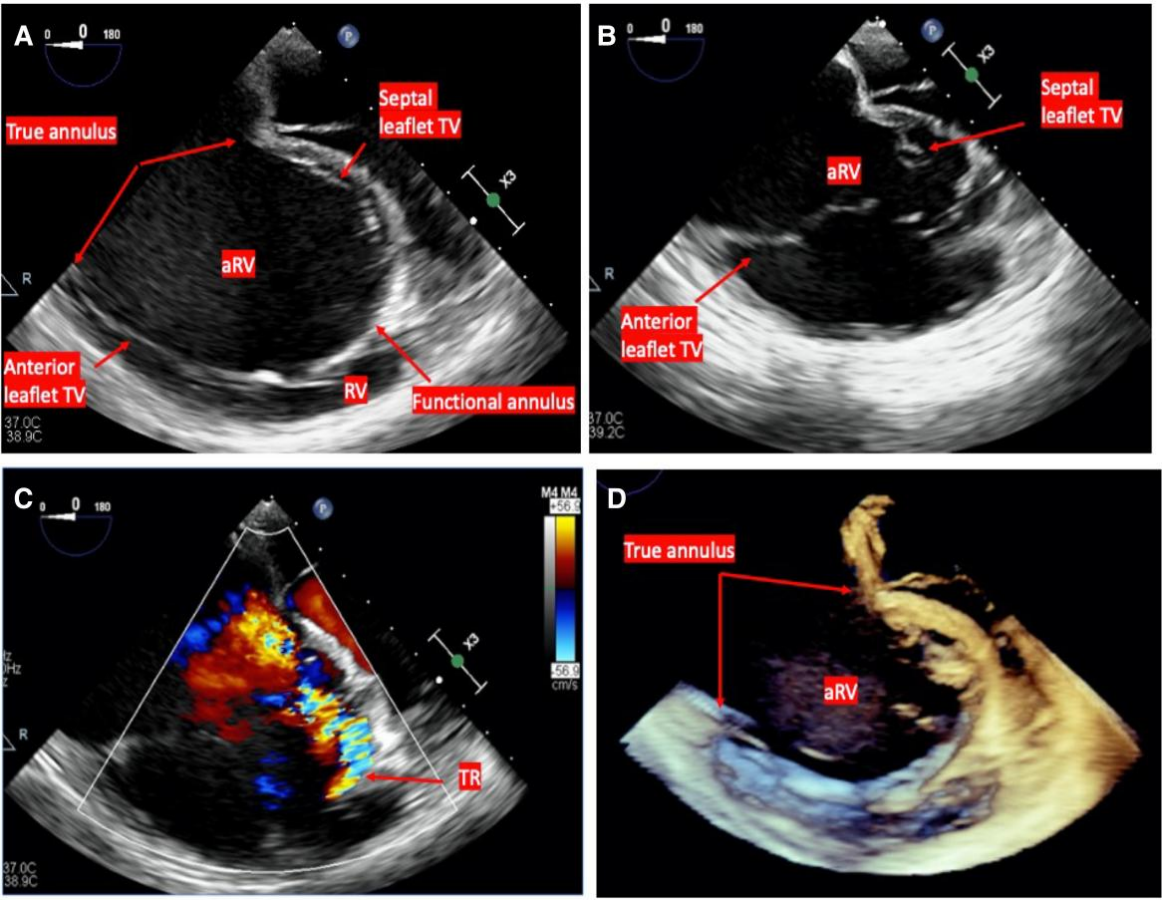
Mid-oesophageal four-chamber view on transoesophageal echocardiography; small right ventricular cavity with leftward septal shift of ventricular septum; the atrialized inlet of the right ventricle is between the true annulus and the functional orifice in diastole (*A*) and systole (*B*). Severe tricuspid regurgitation through the functional orifice (*C*). 3D imaging with dilated atrialized inlet of the right ventricle (*D*). RV, right ventricle; aRV, atrialized inlet of RV; TV, tricuspid valve; TR, tricuspid regurgitation.

**Figure 3:**
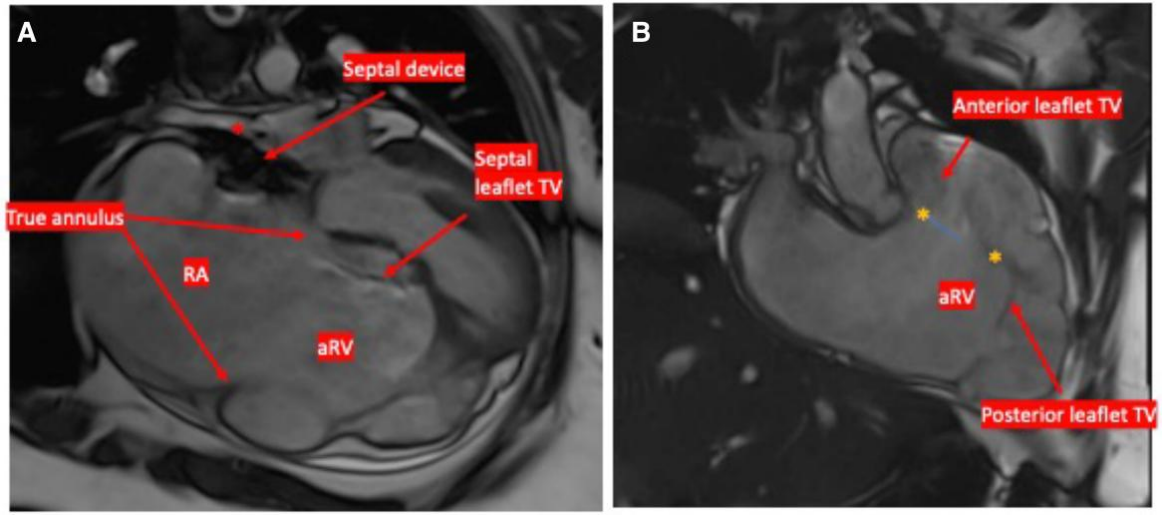
Cardiac magnetic resonance imaging four-chamber view (*A*) and RV inflow–outflow view (*B*) in end diastole demonstrating dilated atrialized inlet of the right ventricle with small functional right ventricle; the distance between the true tricuspid valve annulus and functional tricuspid valve orifice (in yellow asterisks) defines the atrialized inlet of the right ventricle, compressed right lower pulmonary vein denoted by the star (*) next to the septal occlusion device. RA, right atrium; RV, right ventricle; aRV, atrialized inlet of RV; TV, tricuspid valve.

**Figure 4:**
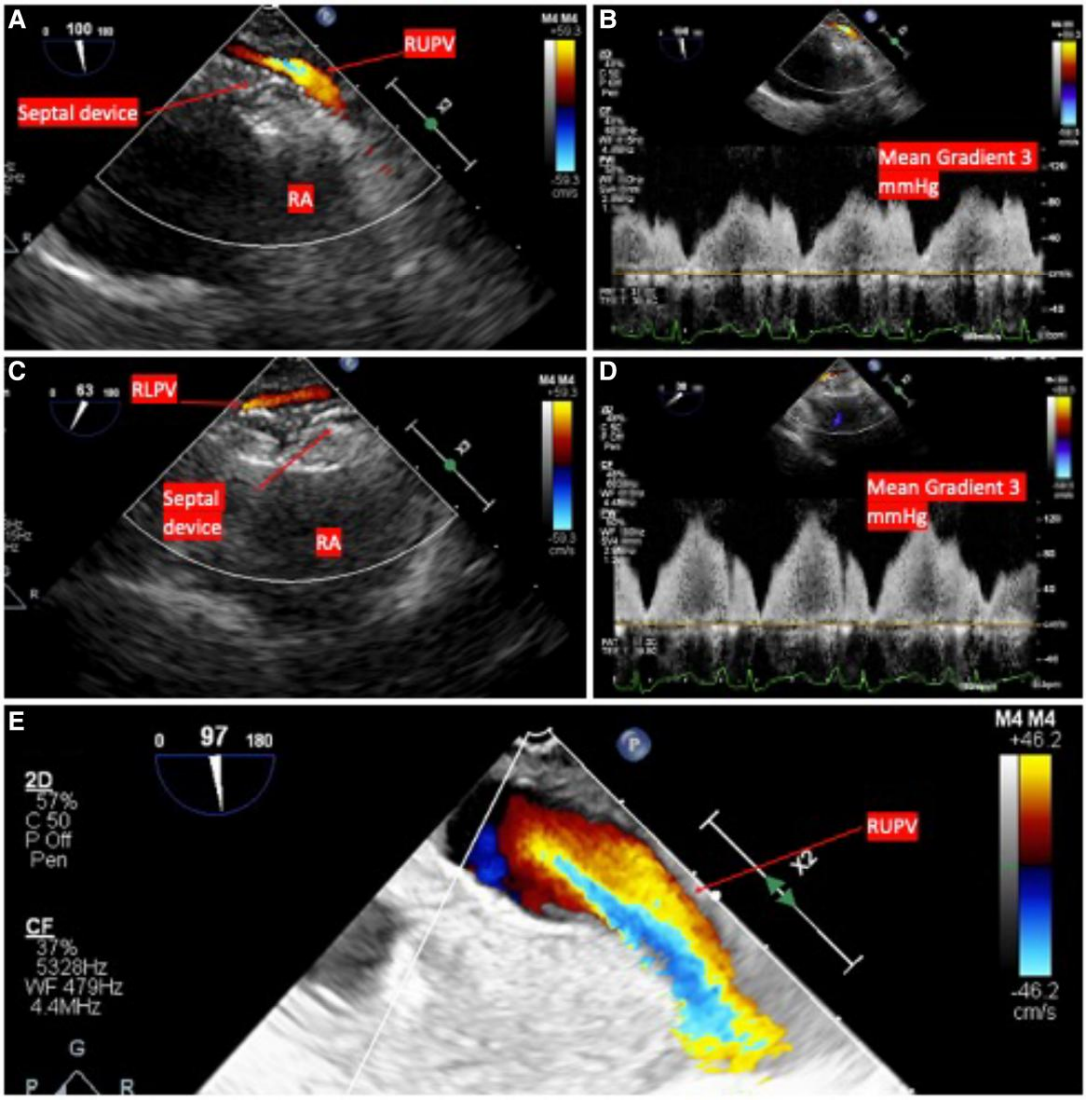
Bicaval view on transthoracic echocardiography. (*A* and *B*) The right upper pulmonary vein and (*C* and *D*) the right lower pulmonary vein, both with colour Doppler and continuous wave Doppler with a mean gradient of 3 mmHg with the septal occlusion device in place. (*E*) The right upper pulmonary vein with improvement in size and flow after the device was removed. RA, right atrium; RUPV, right upper pulmonary vein; RLPV, right lower pulmonary vein.

A 32-year-old woman with a history of unknown congenital heart disease (CHD) presented with progressive dyspnoea. On assessment, she was normotensive with borderline tachycardia and normal O_2_ sat 95%. Neck veins were distended with estimated central venous pressure at 12 cm H_2_O. Cardiac auscultation revealed high pitch S1, systolic click followed by soft systolic murmur heard at the left lower sternal border, and S2 with variable split but accentuated P2. Electrocardiogram (EKG) revealed sinus rhythm with right bundle branch block and right atrial (RA) enlargement and no signs preexcitation (*Figure 1*). The patient reported a history of transcatheter closure of secundum ASD at 19 years old. She had multiple atrial flutter episodes that required multiple emergency room presentations. Tricuspid valve (TV) malfunction was considered based on the presence of systolic click and the low-pitched systolic murmur. Also, considering patient’s known history of ASD and associated atrial arrhythmia, EA was entertained. However, the presence of accentuated P2 was atypical for EA. The other consideration was underlying pulmonary hypertension (PHTN) related to previous long-standing ASD. Transthoracic echocardiography (TTE) and transoesophageal echocardiography (TEE) revealed elongated and redundant anterior TV leaflet with multiple fenestrations, tethering, and apical displacement of the septal leaflet of 10 mm/m^2^ resulting in apical displacement of the functional TV annulus, severely tethered posterior leaflet, dilated true TV annulus = 6.2 cm, and poor leaflet coaptation causing severe TR. There was a compression of the right pulmonary veins (rPVs) by the ASD device (Amplatzer septal occlude; Abbott, St. Paul, MN), no residual intracardiac shunting was noted, and the ASD device was well sealed in the atrial septum. Further evaluation by cardiac magnetic resonance revealed tricuspid regurgitation fraction (RF 43%), dilated functional right ventricle (RV) (right ventricular end-diastolic volume 290 mL, indexed right ventricular end-diastolic volume 160 mL/m^2^) with reduced systolic ejection fraction (RV EF 37%), and severely dilated right atrium (RA 82 mL/m^2^) including the atrialized inlet portion of the RV (aRV) with leftward septal shift causing small left atrial cavity (LA 25 mL/m^2^). The Celermajer index (Great Ormond Street Echocardiography, GOSE) was 0.4. No delayed enhancement was seen in the RV or left ventricle (LV) (*Figures 2–4*). Cardiac catheterization revealed an elevated RA pressure of 22/18/16 mmHg, RV of 45/18, and PA pressure of 44/21/33 mmHg. There was a 3 mmHg difference between the right and left wedge pressures at 15 and 12 mmHg, respectively. The cardiac output (CO) was low at 3.6 L/min using Fick calculation. There was a pulmonary vascular resistance of 5 Wood Units (*Table 1*). There was no residual intracardiac shunting noted in the ratio of pulmonary to systemic blood flow (Qp:Qs): 1:1. Vascular challenge test was not performed as the main pathology related to PHTN was considered post-capillary. Considering the potential risk for accessory pathway-mediated arrhythmia given underlying EA, an electrophysiology (EP) study was performed prior to the surgical repair; the patient was inducible for atrial flutter that degenerated to ventricular fibrillation (VF) requiring cardioversion. The working diagnosis was severe EA and PHTN, caused by a combination of a long-standing left to right shunt prior to ASD device closure and now exacerbated by the ASD device causing pulmonary vein compression, compounding the RV cardiomyopathy. Atrial septal defect device compression on the rPV leads to upstream pulmonary venous (PV) congestion with subsequent pulmonary artery (PA) pressure elevation. Surgical repair of TV along with surgical extraction of the ASD device and maze ablation was planned. Prior to surgical repair, diuresis and PHTN therapy (sildenafil and macitentan) were pursued for a few months. She was prescribed a wearable cardioverter defibrillator after the EP study. The patient underwent surgical TV replacement utilizing 33 mm Epic bioprosthetic valve (St. Jude, Minnesota, USA), RA maze procedure, and extraction of ASD device with patch closure for ASD. Follow-up cardiac catheterization 3 months post-repair revealed improvement of haemodynamics, and therefore PHTN therapy was gradually weaned off (*Table 1*). Repeat EP study with ventricular tachycardia (VT) stimulation was not inducible for VT, there was no indication for automated implantable cardioverter defibrillator (AICD), and the wearable defibrillator was discontinued. Despite the potential concern that often accompanies TV replacement in patients with dysfunctional RV, the noticeable clinical and haemodynamic improvement in our patient was likely related to multiple factors including optimizing volume status along with initiation of PHTN therapy prior to the surgical intervention, and we believe the decompression of the pulmonary vein stenosis with subsequent improvement in PA pressures have positively counter balanced the relative afterload increase that may be encountered post-TV replacement.

**Table 1:** Right heart catheterization results

Location	Pre-operative	Post-operative
RA (mmHg) (a, v, m)	22, 18, 16	9, 8, 7
RV (mmHg)	45/18	23/7
RPA/LPA (mmHg)	44, 21, 33	23, 11, 15
PVR (WU)	5	1.4
RPCWP (mmHg)	15	10
LPCWP (mmHg)	12	10

## Discussion

This report describes a rare case of a patient with EA who presented with worsening RV failure and PHTN post-ASD device closure that led to encroachment on the adjacent rPVs. Pulmonary hypertension is often a rare haemodynamic entity in patients with EA and ASD. The combination of RV cardiomyopathy and tricuspid regurgitation often leads to right to left shunting across ASD and hence protecting the pulmonary vascular bed from the high pulmonary flow encountered in ASD patients. Therefore, the presence of PHTN in EA should trigger an evaluation for post-capillary PHTN. We believe that ASD closure in this setting without addressing underlying dysfunctional TV has caused worsening RV function likely related to progressive elevation of RA pressures post-ASD closure with subsequent leftward atrial septal shift and partial compression of the rPVs. This leads to upstream PV congestion and elevated PA pressure and eventually pulmonary vascular remodelling. We present this case as we thought there are important haemodynamic concepts that need to be thought of prior to ASD closure in EA. Although possible compression of adjacent rPVs is routinely evaluated during ASD device closure, long-term detrimental sequelae can be overlooked. We believe that this is a unique report of iatrogenic PHTN developed late in EA patient post-ASD device closure due to progressive increase in RA pressure resulting in leftward atrial septal shift.

Ebstein anomaly is characterized by apical displacement of the septal and posterior TV leaflets.^1^ Secundum ASD is the most common associated defect and occurs in 71% of cases.^2^ In EA, the myocardium of the aRV is often thin and dyskinetic, resulting in RV dysfunction. The presence of ASD often leads to right to left shunting due to underlying RV diastolic dysfunction. Therefore, ASD closure in EA should be preceded with careful evaluation and consultation with an adult congenital heart disease (ACHD) expert to ensure the patient will tolerate the closure given important later implications related to RV failure.^3^ This often requires ASD balloon test occlusion with close monitoring of haemodynamics, particularly CO. Significant elevation of right-sided pressures and reduction in CO should be alarming signs that ASD closure would not be tolerated. We herein report another factor that needs to be considered before transcatheter ASD closure related to long-term detrimental sequelae of ASD closure without addressing dysfunctional TV.

Indications for surgery in EA include progressive fatigue and RV dysfunction, cyanosis, or atrial arrhythmias. The repair commonly includes TV repair or replacement, partial or complete closure of ASD, and RA reduction and may also include RA maze. Bidirectional Glenn shunt could be considered with severe RV cardiomyopathy, and it is important to ensure normal PA pressures prior to considering Glenn anastomosis; hence, diuresis and PHTN management are important in our patient prior to proceeding with surgical repair.

Patients with EA are at a considerable risk for ventricular arrhythmia (VA) and sudden cardiac death at a similar rate to other common forms of CHD such as TV abnormalities.^4^ In a recent report, the origin of VA in patients with EA was found to be related to either the aRV at the base of the RV or related to surgical scars or diseased Purkinje system in patients with prior surgical intervention.^5^ Although programmed VT stimulation may theoretically uncover latent VA substrates in patients with EA who are prone to VA and sudden cardiac death, there are no well-defined risk factors to propose routine programmed VT stimulation study in this population. Our patient was induced for VF/polymorphic VT prior to surgical intervention, which was felt to be related to decompensated cardiomyopathy at the time of the EP study. In conclusion, although transcatheter ASD closure is feasible in EA, it requires careful evaluation. Assessment of adjacent rPVs should be considered. In patients who do not tolerate balloon test occlusion or with concerns of compression on rPVs, a different strategy should be considered including surgical TV repair or replacement with concomitant partial or complete ASD closure.

## Lead author biography

Dr Ahmed Kheiwa is the medical director of Adult Congenital Heart Disease and Assistant Professor of Cardiology at Loma Linda University Medical Center.
